# Design and Implementation of Analog-Digital Hybrid Beamformers for Low-Complexity Ultrasound Systems: A Feasibility Study

**DOI:** 10.3390/bioengineering11010008

**Published:** 2023-12-21

**Authors:** Heechul Yoon, Junseung Kim, Kunkyu Lee, Tai-Kyong Song

**Affiliations:** 1 School of Electronics and Electrical Engineering, Dankook University, Yongin-si 16890, Republic of Korea; heechul.yoon@dankook.ac.kr; 2 Department of Electronic Engineering, Sogang University, Seoul 04107, Republic of Korea

**Keywords:** analog beamforming, hybrid beamforming systems, point-of-care ultrasound, wearable ultrasound, massive-channel systems, ultrasound system design

## Abstract

Low-complexity ultrasound systems are increasingly desired for both wearable, point-of-care ultrasound and high-end massive-channel ultrasound for 3-D matrix imaging. However, the imaging capabilities, including spatial resolution and contrast, could suffer as low complexity systems are pursued, which remains as an unresolved tradeoff. To mitigate this limitation, this study revisits the general structures of analog and digital beamformers and introduces a hybrid approach, referred to as analog-digital hybrid beamforming, to implement efficient ultrasound systems. The suggested hybrid beamforming takes two stages sequentially, where the first analog stage partially beamforms *M*-channel RF signals to *N* sum-out data (i.e., *M*-to-*N* beamforming), and the second digital stage beamforms *N* partial sums to single final beamformed data (i.e., *N*-to-1 beamforming). Our approach was systematically designed and implemented with only four major integrated circuits, which was capable of driving full 64-channel transmission and reception. The developed system was demonstrated with a customized 64-channel 1-D phased array using a commercial tissue mimicking phantom. From the phantom imaging results, signal-to-noise ratio, contrast-to-noise ratio, and full beam width at half maximum values were quantitatively evaluated. The demonstrated results indicate that the analog-digital hybrid beamforming can be applied to any type of array for sophisticated 3-D imaging and tiny wearable ultrasound applications.

## 1. Introduction

Medical ultrasound imaging has been one of the essential screening and diagnostic tools in the clinic with a wide range of system families from wearable, portable point-of-care ultrasound (POCUS) to high-end sophisticated machines. Wearable ultrasound and POCUS have been increasingly used, as they are relatively easy to learn, accessible to patients, and versatile as a diagnostic tool [1,2]. Moreover, due to recent technical advances, ultrasound systems have become more functional, capable of offering acoustic, biomechanical, and optical properties of complex pathologies in real-time [3,4,5]. Therefore, clinical utility of ultrasound also has expanded. In addition to cardiac and thoracic applications, POCUS can be useful to patients with dyspnea, hemodialysis, pneumonia, and ventricular dysfunction [6].

The diagnostic capabilities of ultrasound imaging with wearable ultrasound, POCUS, and even high-end machines basically rely on spatial resolution and contrast of the image [7,8]. However, ultrasound waves diffract along the propagation, and thus, the resolution of ultrasound is only favorable near the focus [9]. This limitation, because of wave diffraction in imaging resolution, has been continuously improved with digital techniques, including dynamic receive beamforming [10,11,12]. Synthetic aperture ultrasound, ultrasound localization microscopy, referred to as super-resolution ultrasound, and many other approaches reducing the level of sidelobe and the beam width of main lobe have been investigated to enhance the spatial resolution [13,14,15,16].

This fundamental limitation on the spatial resolution of ultrasound is associated with electrical focusing of an array transducer; an array with a larger dimension with a greater number of active channels for transmit/receive operations can improve the spatial resolution in general [17]. For this electrical focusing, digital beamforming is most typically used in modern ultrasound scanners, which require one analog-to-digital converter (ADC) and one digital first-in-first-out (FIFO) memory per receiving channel [11]. Therefore, the greater number of channels linearly increases the system complexity and thus the production cost. This would be critical not only for a small, handheld device for wearable ultrasound and POCUS, but also for high-end ultrasound supporting 3-D imaging applications [18,19,20]. Recent progressive utilization of a 2-D matrix array necessitates one-to-one connection of every array element and system channel, which is often a major concern in its practical implementation. For example, a 32 × 32 2-D matrix array simply requires 1024 channels with the same number of pulsers and receivers [18]. Thus, to reduce the active number of channels in 3-D imaging, sparse arrays and row-column addressed arrays are often considered, but at the expense of the image quality [21,22,23].

Adopting analog beamforming or analog-digital hybrid beamforming approaches could help reduce the amount of system components [24]. As the analog beamformer sums out receiving analog signals without digitization, it does not use ADCs and digital FIFOs for every channel but uses the analog FIFO simply consisting of one capacitor per FIFO cell [25,26]. Here, the analog FIFO cells are serially connected to continuously store receiving signals within a given time resolution of the system (typically a few nanoseconds). With the smaller time resolution, receiving delays for beamforming can be more accurately controlled. The other important aspect of the analog beamforming is the total length of the analog FIFO cells, which determines the maximum delay range among receiving channels [26]. However, every cell in the analog FIFO should be precisely time-controlled for dynamic receive beamforming or data loss can occur over time due to the capacitor discharging. Thus, when the time delay differences between receiving channels are long, the data loss can become more problematic.

Analog-digital hybrid beamformers could overcome these respective downsides of both analog and digital beamformers [27]. To mitigate the data discharging problem in the analog FIFO cell and to leverage the system complexity, this study investigated a two-stage analog-digital hybrid beamformer architecture and its systematic implementation. The first analog part of the beamformer focused the *M*-channel receiving signals to *N* partial sum out signals (i.e., *M*-to-*N* beamforming), which were digitized for the second digital beamforming stage. The second digital part beamformed *N* signals to one final sum out (i.e., *N*-to-1 beamforming). Design and implementation of our two-stage analog-digital hybrid beamforming approach with phantom imaging results were presented using a customized 1-D phased array, however, the same principles validated in this study can be applied to any type of array, including a 2-D array transducer with a greater number of channels in general.

## 2. Materials and Methods

### 2.1. Digital and Analog Beamformers

**Figure 1:**
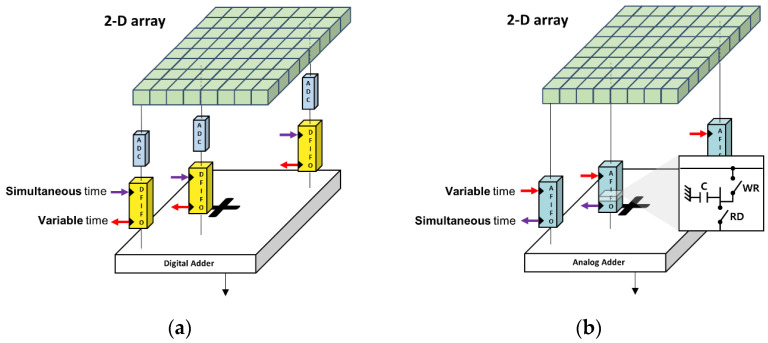
General structures of (**a**) digital and (**b**) analog beamformers. A digital beamformer generally consists of an ADC and a digital FIFO for every channel, followed by a digital adder for final beamformation. An analog beamformer does not have ADCs, but uses analog FIFOs comprising a single capacitor and two switches for read/write operations per receiving channel, followed by an analog adder.

Figure 1 illustrates digital and analog beamformer structures. As shown in Figure 1a, a digital beamformer generally comprises one ADC and one digital FIFO per receiving channel and a digital adder for final summation of all delayed signals [11,12]. For the digital beamformer, all receiving echoes were stored in every digital FIFO synchronously, which were read out at different variable times, depending on the time delays required for coherent summation [10]. The digital beamformer was expected to be precise in beamforming and once the RF data was sampled and stored in the FIFO, the data was stably utilized for beamforming. However, because one ADC was required for the individual receiving channel for digital beamformers, the system complexity and associated power consumption linearly increased with respect to the number of channels.

Analog beamformers, as shown in Figure 1b, could resolve the challenges in digital beamformers, but have other limitations. Each cell in the analog FIFO has two switches controlling write and read operations of received analog signals and a capacitor for their storage [26]. In contrast to digital beamformers, analog beamformers apply focusing delays prior to deposition of the data into the FIFO. Thus, receiving ultrasound echoes are stored at variable different times along the analog FIFO cells, depending on the time delays for beamforming based on a sample-and-hold approach. For the final beam formation, the stored data is read out simultaneously, possibly followed by a simple wired addition. Although analog beamformers can make the imaging systems more efficient, the data loss in the cells is an issue. The amount of data loss between channels can vary because of focusing delays, which can cause nonuniform signal strengths between channels. Furthermore, the number of taps (or cells) of analog FIFO determines the longest focusing delays between channels, limiting the utility of the imaging systems.

### 2.2. Analog-Digital Hybrid Beamformer

**Figure 2:**
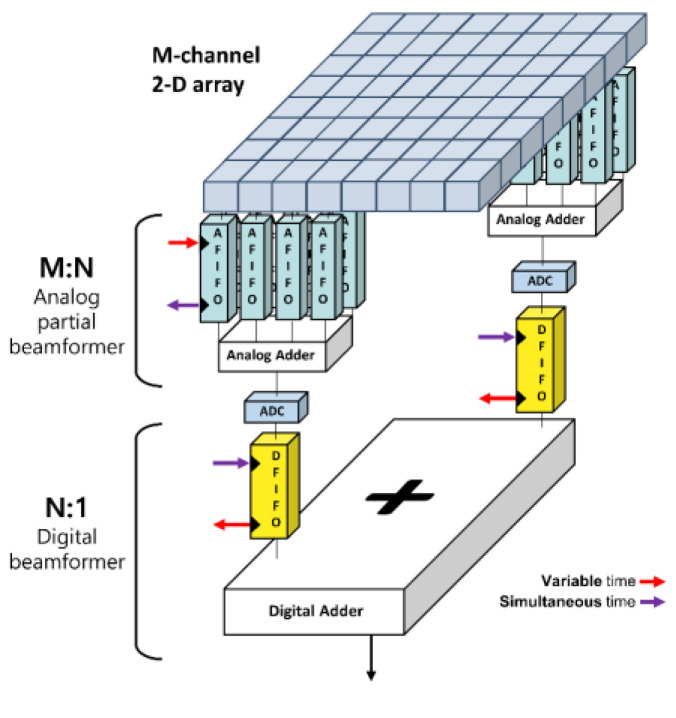
Analog-digital hybrid beamformer for massive-channel ultrasound systems. Any type of array with an *M* number of elements beamform the first analog signals, producing *N* partially beamformed signals, which are digitized for the second digital beamforming for final delay-and-sum.

An analog-digital hybrid beamformer could leverage respective limitations in analog and digital beamformers [28]. As shown in Figure 2, given an *M*-channel array transducer, *N* analog beamformers first partially focus analog RF data and output *N* partial sum data (i.e., *M*-to-*N* analog beamforming), which is sampled through ADCs and digitally beamformed (i.e., *N*-to-1 digital beamforming), producing a single sum-out result. In the *M*-to-*N* analog beamforming stage, a total of *M* channels are grouped into *N* sub-aperture groups. Thus, each analog beamformer takes M/N channels of RF data and produces *N* partial sums, followed by the *N*-to-1 digital beamforming process.

**Figure 3:**
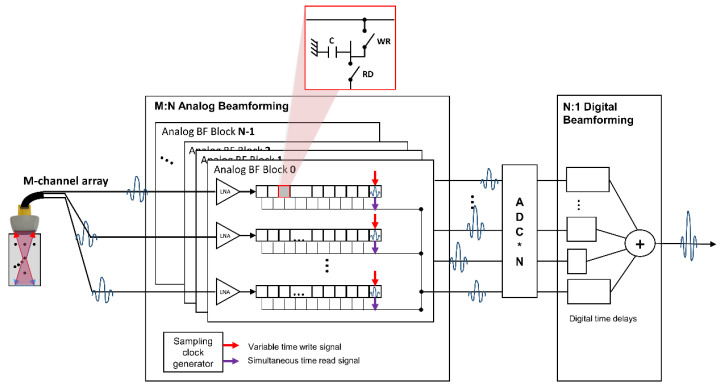
System block diagram of an analog-digital hybrid beamformer with an *M*-channel array transducer. The analog beamforming part produced *N* beamformed signals from *M* receiving RF data, which were sampled then via *N* ADCs for the last *N*-to-1 digital beamforming. Here, the sampling clock generator controlled the data charging timing of each capacitor in the serial taps in the analog FIFO. ADC * N in the figure means that N number of ADCs.

Figure 3 shows the block diagram of the analog-digital hybrid beamforming system. Each cell, referred to also as a tap, in the analog FIFO, consisted again of a single capacitor with two write/read switches. These taps (or cells) are serially connected to support continuous storing of the receiving signals and their beamforming, which is controlled by the sampling clock generator with variable write and simultaneous read timing signals. Partial analog sums of *N* analog partial beamformers are sampled with *N* ADCs and digitally beamformed at the end.

**Figure 4:**
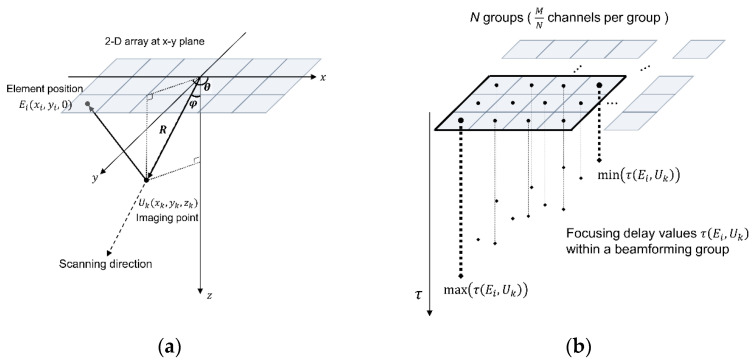
(**a**) Geometrical representation of a general 2-D array in the 3-D space and (**b**) receiving delay calculation with a sub-group of 2-D array. Scanning direction was defined with polar and azimuthal angles (i.e., θ and φ) and the imaging point-of-interest was defined as $U_{k}(x_{k},y_{k},z_{k})$ in (**a**). Given sub-groups of partial analog beamforming in (**b**), the maximum and the minimum receiving time delays should be within the limit of the analog beamformer, which was determined by the number of the taps (or cells) in the analog FIFO.

Geometric representation of a 2-D array transducer and delay calculation for partial analog beamforming are described in Figure 4. The general 2-D array with *M* elements was assumed to be located at the x-y plane, as shown in Figure 4a. The positions of each array element and the imaging target are represented by $E_{i}(x_{i},y_{i},0)$ and $U_{k}(x_{k},y_{k},z_{k})$, respectively, in the 3-D coordinate system. Here, the index *i* represents individual elements ranging from 1 to *M* and the index *k* represents the imaging points. θ and φ are polar and azimuthal angles defining the scanning direction. *R* represents the distance between the array origin and the imaging point of interest. The two-way round-trip time-of-flight $\tau \left(E_{i},U_{k}\right)$ can then be defined as follows:

(1) $$\tau \left(E_{i},U_{k}\right)=\left(R+\sqrt{(x_{k}-x_{i}{)}^{2}+(y_{k}-y_{i}{)}^{2}+z_{k}^{2}}\right)/c$$

where *c* represents the speed of sound. Equation (2) calculates the round-trip delays from all 2-D elements for dynamic beamforming. The origin of the array was used to form all sectored scan lines for phased-array imaging in this study. Considering receiving delays for a sub-group with M/N channels, as described in Figure 4b, the number of taps of analog FIFOs should support the maximum and minimum delays required within a sub-group, as the sub-group elements continuously receive and store the analog data for beamforming. Specifically, defining the sample-and-hold time of each tap as $T_{s}$, also referred to as a tap charging time, a maximum delay difference within the sub-grouped elements should satisfy the following condition.

(2) $$\max\left(\tau \left(E_{i},U_{k}\right)\right)-\min\left(\tau \left(E_{i},U_{k}\right)\right)\leq the\;number\;of\;taps\times T_{s}$$

The condition in Equation (2) guarantees that the partial summation of the analog part of the beamformer can rely on the receiving RF data from the current target-of-interest. If this condition is not satisfied, the data received from the different locations can be undesirably mixed during beam formation. Every subgroup of partial analog beamforming should satisfy Equation (2).

### 2.3. Design and Implementation of the Analog-Digital Hybrid Beamforming System

**Figure 5:**
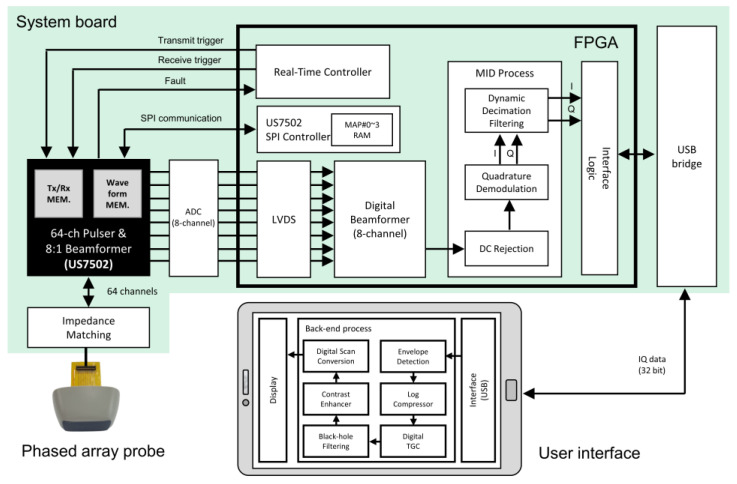
Functional block diagram of the implemented system consisting of four major ICs—two AFE ICs for analog beamforming and sampling (US7502 and AFE5801), one FPGA (Artix-7 XC7A200T), and a MCU (CYUSB3014-BZXI) for USB interface.

Figure 5 shows the functional block diagram of our analog-digital hybrid beamforming system, consisting of a field programmable gate array (FPGA), analog front-end (AFE) integrated circuits (ICs), and a microcontroller unit (MCU). For the AFE part, US7502 (ABLIC, Inc., Tokyo, Japan) was used for 64-channel transmission and 64 to 8 partial analog beamforming. The US7502 IC had the waveform and delay memory for transmit focusing, allowing use for electric focusing of 64-channel ultrasound pulses. The 8-channel analog beamformed data from the US7502 IC was digitized through a single AFE IC with 8-channel ADCs (AFE5801, Texas Instruments, Dallas, TX, USA) and then transferred to the FPGA (Artix-7 XC7A200T, Xilinx, Inc., San Jose, CA, USA) through low-voltage differential signaling (LVDS) for digital beamforming and signal processing. A tablet host device (Galaxy Tab S6, Samsung Electronics Co., Ltd., Suwon-si, Republic of Korea) and the developed system were interfaced via universal serial bus (USB) communication through the bridge MCU (CYUSB3014-BZXI, Infineon Technologies AG, Neubiberg, Germany). Thus, only four major ICs (two AFE ICs, one FPGA, and one MCU) were used to design an ultrasound imaging system capable of full 64-channel driven transmission and reception.

To suppress the DC noise signals in the system board, analog high-pass filters in the US7502 IC were used, which was followed by the digital DC rejection in the FPGA of the system after digital beamforming. The US7502 IC performed analog partial beamforming, producing 8-channels of beamformed data, which were digitally delay-and-summed for complete beamforming. For the digital beamforming, the common delay value for the 8-channel partial sum-outs was taken from the minimum sub-group delays obtained in Equation (2). The post-beamformed RF data processed in the FPGA was demodulated to provide the IQ data, which was transferred to the host device for further signal and image processing to reconstruct B-mode ultrasound images in real time. The final sum-out data from the digital beamformer was filtered to mitigate any further DC components. For the demodulation part, we used the quadrature demodulation scheme with look-up-table-based cosine and sine values sampled at the 50-MHz system clock rate. For low-pass filters to remove high frequency components, their filter coefficients were loaded from the host device to the registers in the FPGA and the dynamic decimation process was followed to keep the number of data samples consistent. From the IQ data, the envelope of the signal was first extracted and then log-transformed to adjust the dynamic range of the signals. Digital time gain compensation along the depth was applied to the log-transformed data to recover the attenuated intensity of the signals. A simple 3 by 3 median filtering was applied to fill the black holes in the gain compensated data to mitigate the speckle noises. A final signal processing step in the host device was the digital scan conversion, which reformatted the image to display a sector form of the images obtained via the phased array steering from −45° to +45°.

For every scanline with varying steering angles, the FPGA delivered a set of parameters with transmit pulse delays and received dynamic focusing delays to the internal memory in the US7502 IC through the serial peripheral interface (SPI) communication, as shown in Figure 5. Moreover, whenever focus or view depth were updated from the host device, new delay values were calculated and set to the US7502 IC for dynamic beamforming. The transmit trigger was issued after the SPI communication was completed for all delay settings, followed by the receiving trigger to initiate the dynamic beamforming process. 

**Figure 6:**
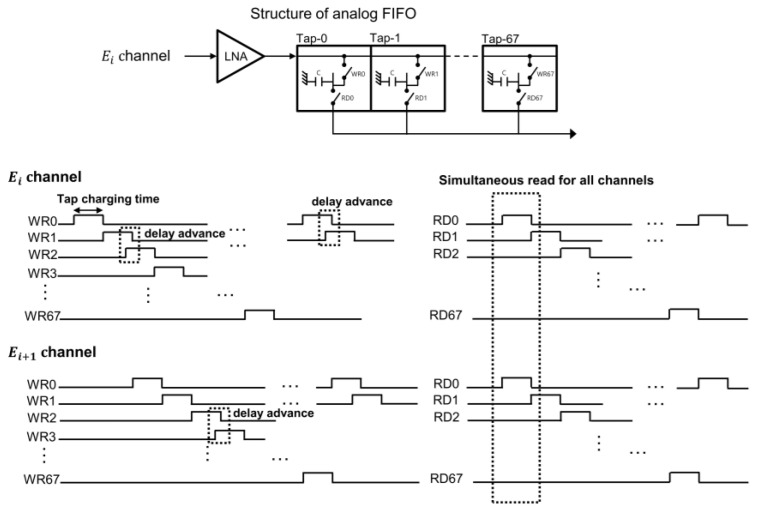
Analog FIFO structure with 68 taps and associated write/read timings in each channel. WR0 to WR67 control signals stored the analog data in the corresponding cell of the analog FIFO in each channel. $E_{i}$ and $E_{i+1}$ channels in the figure meant that the different positions of the array elements induced delayed write timings. Writing receiving analog signals between cells (or taps) can be overlapped by the delay advance time. Read-out operations with RD0 to RD67 control signals were consecutively performed along the cell.

A custom one-dimensional 64-element phased array was specifically used in this study, but there is no limitation in the different number of elements or the transducer types. In other words, our approach can be extended to other types of transducers with greater number of elements such as a 2-D matrix array. As shown in Figure 6, each channel of the analog beamformer in the US7502 had 68 taps with 20 ns of a tap charging time ($T_{s}$), as the clock rate of the overall system was at 50 MHz. In other words, incoming pre-beamformed analog RF signals in each channel can be continuously stored along the 68 analog FIFO taps at every 20 ns. Write control signals (WR0 to WR67) indicate serial charging of each capacitor in the 68 taps from Tap-0 to Tab-67 per channel, as shown in Figure 6. Here, for more precise beamforming, storing the signals in the neighboring taps can be overlapped with a 5 ns time step, which was referred to as a delay advance. The maximum number of this delay advance event was limited to 255 in the US7502 IC for the entire firing, and the next delay advance from the prior event happened at 8$T_{s}$, 16$T_{s}$, 64$T_{s}$, 256$T_{s}$, and 1024$T_{s}$. The delay advance timing steps and the number of delay advance events were all specified by the IC manufacturer. After storing the analog signals correctly in the relevant taps in the analog FIFO with appropriate beamforming delays, reading and summing operations were conducted simultaneously at every 20 ns, which finally produced partially beamformed 8-channel analog data.

### 2.4. Phantom Imaging Experiments

A custom-made 64-element phased array transducer with a 0.24 mm pitch (i.e., element spacing), elevationally focused at 80 mm was used to verify the implemented system. A single cycle ultrasound pulse at a center frequency of 3.2 MHz was induced to the array transducer for imaging a commercial tissue mimicking phantom with cysts and point targets (Model 054GS, Mirion Technologies Inc., Atlanta, GA, USA).

To assess the imaging capabilities of the system, a signal-to-noise ratio (SNR) and a contrast-to-noise ratio (CNR), with lateral beam widths at half the maximum of the signal were measured on pin and cyst targets of the phantom, respectively. SNR and CNR values were evaluated via the following equations [29,30]:

(3) $$SNR=20{log}_{10}\frac{\left|\mu_{s}\right|}{\left|\mu_{n}\right|}$$

(4) $$CNR=20{log}_{10}\frac{\left|\mu_{s}-\mu_{b}\right|}{\left|\sigma_{s}^{2}+\sigma_{b}^{2}\right|}$$

where $\mu_{s}$ and $\mu_{n}$ in Equation (3) were the averaged signals on the point target and surrounding speckle noise region, respectively. $\mu_{s}$ and $\mu_{b}$ in Equation (4) were the averaged signals from the foreground and the background regions of cysts in the phantom, respectively. $\sigma_{s}$ and $\sigma_{b}$ were the standard deviations evaluated from the same regions, respectively.

## 3. Results and Discussion

### 3.1. Imaging Results from a Phantom with the Integrated System

**Figure 7:**
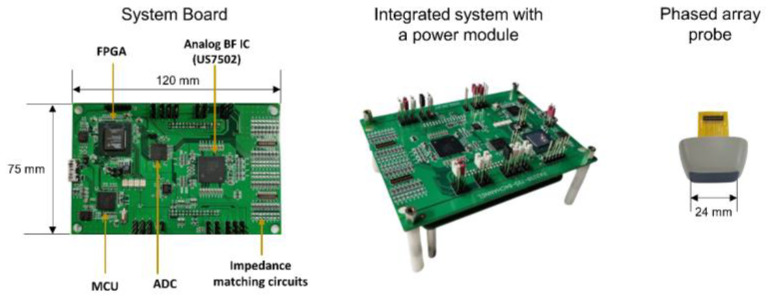
Implemented systems with four major ICs (a FPGA, a MCU, and two AFE ICs) for analog-digital hybrid beamforming and a custom 64-element phased-array used in this study. The main system was built in a printed circuit board with a dimension of 74 mm by 120 mm, whose power sources were supplied via the power module attached to the bottom of the main system board. A customized 64-element phased array transducer can be connected to the system board with impedance matching circuits in between.

The integrated system with a power module connected to the bottom of the main system board is shown in Figure 7. The power module, which is not covered in this paper, provided stable high voltage sources of ultrasound pulsers for transmission in the AFE (i.e., US7502 IC) and several low voltage sources for operating two AFE ICs, a FPGA, a MCU, and other peripheral ICs and components in the system board. The system clock rate for the FPGA and the AFE ADCs was at 50 MHz and another clock rate of 200 MHz was also used for precise delay advancing in the analog partial beamformer IC, which resulted in a 20 ns tap charging time and a 5 ns delay advance time, respectively, as described in the previous section.

**Figure 8:**
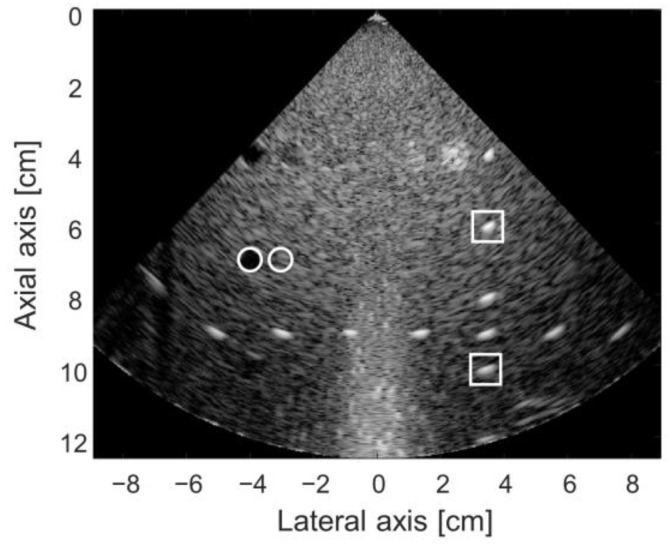
Reconstructed B-mode image with a 12 cm view depth obtained from the tissue mimicking phantom using the implemented system in this study. White circles and rectangles in the image were respectively used for CNR and SNR values. The CNR value measured from the cystic target and the corresponding background was 3.5 dB and the SNR values measured at two pin targets with different depths were 27.6 dB and 35.6 dB, respectively.

For B-mode ultrasound imaging demonstration, a single-cycle pulse with a 3.2 MHz center frequency was applied to a custom-made phased array transducer with 64 elements. The elevational focus of the probe was at 80 mm. The acoustic transmit focus was electrically created at a 60 mm depth with a total of 128 scanlines. The field of view of the image was created with −45° to +45° of scanning angles. Analog partial beamformed data (8-channel data form 64 channel inputs) from the US7520 AFE IC was transferred to the FPGA for the remaining digital beamforming after digitization via the ADCs in the AFE5801 IC. Post-beamformed IQ data was then processed in the FPGA, which was transferred to the host tablet device for further signal and imaging processing before displaying the image in real-time. The final B-mode image as shown in Figure 8 was captured from the host tablet and visualized with MATLAB R2023a software offline.

The SNR values from two pin targets (white boxes in Figure 8) were evaluated. The SNR value of the pin target located near the acoustic focus was 35.6 dB and that of the deeper target was 27.6 dB, respectively. From these two pin targets, we also measured the lateral beam widths, which were 4.1 mm and 5.1 mm, respectively. The CNR value from the hypoechoic cyst and nearby background (white circles in Figure 8) was 3.5 dB. In Figure 8, the white noise signals at the middle-bottom of the image were introduced when the time delays are close in our system, which needs to be improved in the future version of the system. Supplementary video clip captured on our system operating real-time can be found in Video S1.

### 3.2. Practical Considerations in Analog Beamforming

Each analog FIFO per receiving channel for partial analog beamforming in the US7502 IC had 68 taps. As 8-to-1 partial beamforming was conducted in this study, we needed to consider whether all delayed signals coming into each subgroup of 8 channels can be reliably stored within a 68-tap length under the given imaging condition. In other words, a time difference between the maximum and minimum delays calculated in each subgroup elements for any imaging point for beamforming should satisfy Equation (2). In our study, all sub-group elements were consecutive elements in a row to minimize the delay differences in elements in each sub-group. As the tap charging time $T_{s}$ of our system was 20 ns and each channel had 68 taps in the analog FIFO, a maximally allowed time delay difference between channels in each subgroup was 1360 ns (=68 taps × 20 ns). Thus, when the steering angle becomes large for phased array imaging, a delay difference within the 8-channel group may exceed 1360 ns, which needs to be avoided for reliable beamforming. For example, when θ = 0°, φ = −45°, and *R* = 5 mm, the time delays for the first and eighth elements of the array used in this study were 9087 ns and 7570 ns from Equation (1), which means that this imaging condition made the delay difference longer than 1360 ns and this case cannot be supported for beamforming with our system.

**Figure 9:**
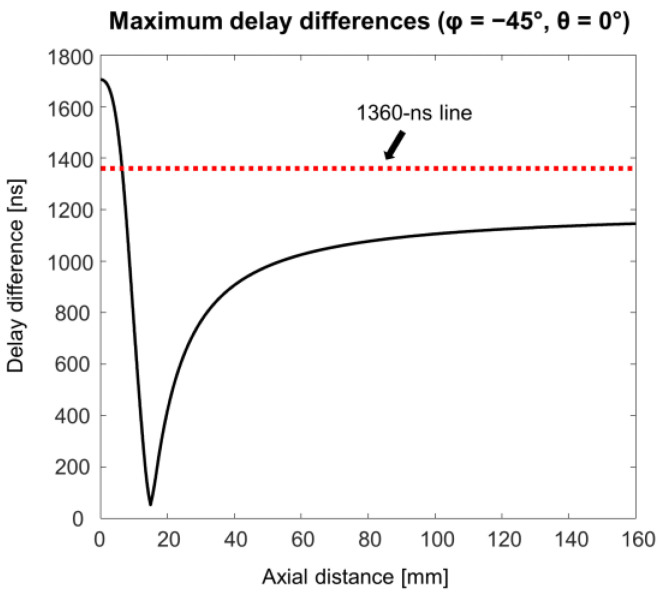
Maximum delay differences over the axial distance for the first-grouped elements (i.e., from element 1 to element 8) with −45° of a steering angle φ in the 1-D phased array, which demonstrates that the near field signals by about a 6 mm depth cannot be correctly beamformed with these imaging conditions, as the time delay difference exceeds the system limit (i.e., 1360 ns).

From the worst-case analysis with our system specifications, we found that 93 taps and 366 delay advances were required for sector format imaging of a 16 cm depth, which was clearly beyond the system capability; the number of taps and the maximum number of delay advances in the US7502 IC were required to be less than or equal to 68 and 255, respectively. Figure 9 shows the maximum delay differences along the depth in the first-grouped elements (i.e., the first to eighth elements) in the array when the scanline was maximally steered at −45°. As indicated by the 1360 ns line (a red dotted line) in Figure 9, the imaging samples from 0 mm to 6 mm cannot be correctly beamformed. Thus, based on this analysis, near field data by 6 mm was ignored to avoid missing signals during the partial analog beamforming process, which was applied to reconstruct the final image presented in Figure 8.

### 3.3. Comparative Evaluations on Beamforming Archtectures

**Figure 10:**
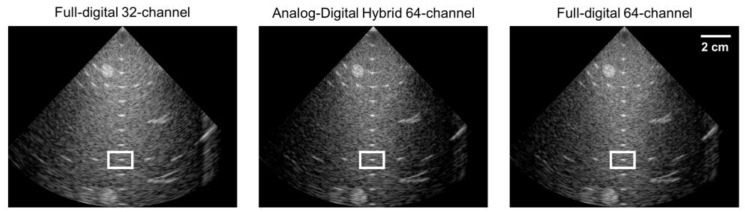
Reconstructed B-mode images with three different approaches—32-channel full digital beamforming, 64-channel virtual analog-digital hybrid beamforming, 64-channel full digital beamforming. White rectangles in the images indicate the region-of-interest to measure the lateral resolution.

As our custom-built system is dedicated to performing analog-digital hybrid beamforming, to further demonstrate the capability of our approach over full digital beamforming, which is gold standard in current ultrasound imaging systems, we utilized an ultrasound research platform (Vantage 128, Verasonics Inc., Kirkland, WA, USA). The 64-channel analog-digital hybrid beamforming introduced in this paper was virtually implemented offline on 64-channel digital RF data with the same time delays applied in the analog partial beamforming stage. Thus, the total delay was limited to 1360 ns, which was given by 68 taps in the analog FIFO and 20 ns of the tap charging time. Time shifts in each channel were also controlled with a 5 ns step. As a result, different beamforming architectures could be compared using the same platform, which allows us to avoid numerous system-dependent parameters, including signal-to-noise ratio, pulsing methods, imaging/beamforming algorithms, etc. Figure 10 shows phantom imaging results reconstructed with three methods with two full digital approaches and a virtual analog-digital hybrid approach.

**Figure 11:**
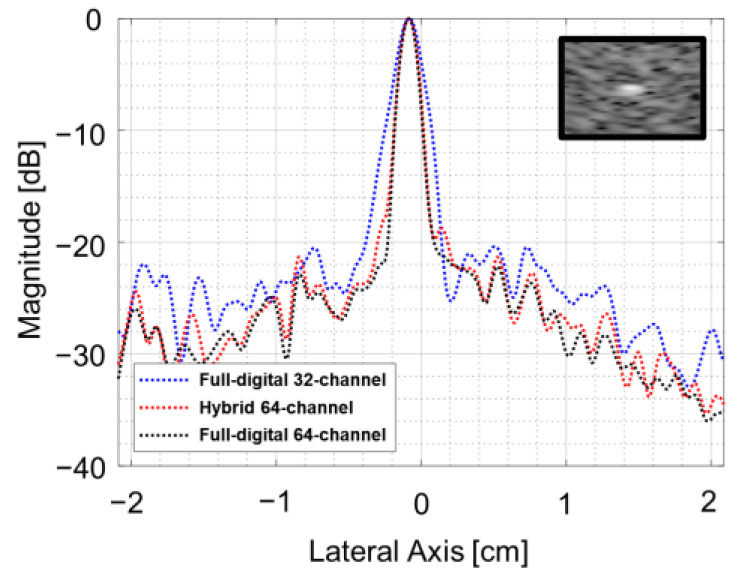
Lateral beam profiles measured on a point target from the phantom experiments using a Verasonics system.

The analog-digital hybrid approach in Figure 10 shows the improved lateral resolution over the full digital beamforming with 32 channels and the similar qualities over the full digital beamforming with 64 channels except the small degradations in near field regions. This resulted from the fact that the delay difference longer than 1360 ns was not supported in the analog partial beamforming stage, which was applied to the virtual implementation for comparative evaluation. Figure 11 shows the lateral beam profiles measured on a point target in white rectangles in Figure 10, indicating that the lateral resolutions of the analog-hybrid beamforming and the full digital beamforming with the same number of channels were almost the same, as expected in the imaging results in Figure 10.

Potential costs for ICs used in 64-channel ultrasound systems with two different approaches—full-digital and analog-digital hybrid approaches are listed in Table 1. Here, we excluded FPGA, MCU, and other power ICs assuming that they are common for use in both architectures. For the 64-channel ultrasound system, the total costs in Table 1 suggest that the hybrid approach can be more economic. This benefit of the hybrid system would further increase if a larger number of channels were considered.

Resource utilization in the Artix-7 FPGA for partial digital beamforming and other common modules in our approach extracted from the development tool (Vivado, Xilinx, Inc., San Jose, CA, USA) is summarized in Table 2. Because of the low complexity of beamforming in our approach, the resources used for beamforming remain relatively low. Furthermore, other modules for quadrature demodulation and data communication were independent of the number of channels. Thus, if our approach is applied to more complex 2-D or 3-D imaging applications, the resources only for digital beamforming-related modules, including delay calculation and channel memory, would linearly increase with respect to the number of channels. However, for full digital imaging systems, the resources for the beamforming part in Table 2 would be eight times more in the 64-channel case.

### 3.4. Limitations and Future Directions

This study has designed and implemented an analog-digital hybrid beamforming system with the imaging demonstration on a tissue mimicking phantom. In theory, as described in the previous sections, in terms of power consumption and system complexity, the full analog beamformers outperform the fully digital beamformers, and thus, the hybrid approach should be in between. For the beamforming accuracy and reliability, the digital beamforming would be the best, as no data loss in the capacitor cells and no delay limit were expected. However, this feasibility study on the hybrid beamforming system did not quantitively evaluate its capabilities over full digital and full analog beamformers. Moreover, the design and demonstration on hybrid beamforming were conducted with the PCB-based system using commercially available ICs for proof-of-concept. Our future work includes the design of an application-specific integrated circuit (ASIC) potentially containing all beamforming and AFE functions in a single IC, which is expected to mitigate the system and DC noises. The other limitation of this study is that we have demonstrated the proof-of-concept system with B-mode ultrasound. However, our system architecture does not limit the implementation of other imaging modes in ultrasound, including color Doppler and harmonic imaging with pulse inversion.

## 4. Conclusions

This study revisited the general structures of analog and digital beamformers and compared their advantages and disadvantages in ultrasound beamforming. To mitigate their respective limitations, we have suggested a combined approach, referred to as analog-digital hybrid beamforming, potentially useful for the efficient design of massive-channel ultrasound systems. We designed and demonstrated the imaging system with the reduced resources, capable of 64-channel partial analog-digital hybrid beamforming, which can be used for wearable and POCUS applications and extended to the greater number of channels for 3-D imaging applications.

## Figures and Tables

**Table 1:** Expected IC costs for 64-channel portable ultrasound systems.

IC Lists	Full-Digital 64-ch	Hybrid 64-ch
16-ch pulser (S-UM5587P, ABLIC)	USD 74.9 × 4 (ea)	Not used
8-ch ADC (AFE5801, TI)	USD 76.3 × 8 (ea)	USD 76.3 × 1 (ea)
Partial BF IC (US7502, ABLIC)	Not used	USD 191.2 × 1 (ea)
FPGA, MCU, power ICs	Assumed to be the same (excluded)	
Total cost	USD 910.0	USD 267.5

**Table 2:** Resource utilization for analog-digital hybrid 64-channel ultrasound systems implemented on the Artix-7 FPGA.

Module		Resource Utilization			
		Register		DSP	BRAM
		LUT	Flipflops		
Delay calculation	Delay calculator	2.8%	1.7%	6.5%	0.5%
	Fractional interpolator	3.4%	2.5%	2.3%	-
Channel memory	Digital FIFO	-	-	-	2.2%
Others		5.8%	6.2%	13.5%	13.6%
Total		12%	10.4%	22.3%	16.3%

## Data Availability

Data are contained within the article and Supplementary Materials.

## Supplementary Materials

The following supporting information can be downloaded at: https://www.mdpi.com/article/10.3390/bioengineering11010008/s1, Video S1: Real-time imaging results captured from the system.
